# Supplementary material for: Mechanochemical forces regulate the composition and fate of stalled nascent chains
Source: bioRxiv. 2024 Oct 14:2024.08.02.606406. Originally published 2024 Aug 4. Preprint. [Version 2] doi: 10.1101/2024.08.02.606406 (PMC11312545; doi:10.1101/2024.08.02.606406)

bioRxiv preprint doi: https://doi.org/10.1101/2024.08.02.606406; this version posted October 14, 2024. The copyright holder for this preprint (which was not certified by peer review) is the author/funder, who has granted bioRxiv a license to display the preprint in perpetuity. It is made available under aCC-BY 4.0 International license.

Figure S1 CAT tails have diverse composition, degradation, and aggregation propensities. (A-D) Whole cell IBs of lysates containing model RQC substrates expressed in different strains and gel densities. Cartoon of model RQC substrates with single lysine superfolder GFP (1K-sGFP) indicated where applicable. CAT tails are marked by a red line. Arrowhead (➤) indicates readthrough product formed after bypassing the polyarginine arrest sequence.

bioRxiv preprint doi: https://doi.org/10.1101/2024.08.02.606406; this version posted October 14, 2024. The copyright holder for this preprint (which was not certified by peer review) is the author/funder, who has granted bioRxiv a license to display the preprint in perpetuity. It is made available under aCC-BY 4.0 International license.

Figure S2 CAT tail sequence determines degradation and aggregation. (A-B) Whole cell immunoblots of GFP-20 and GFP-40 expressed in *ltn1Δ* cells with varying CAT tailing capabilities: normal CAT tailing (WT, lane 1), no CAT tailing (*rqc2Δ,* lane 2; *rqc2*-D98A, lane 3), and impaired CAT tailing (*rqc2*-D9A, lane 4). **(C)** Mean stability measurements for GFP-20 and GFP-40 substrates in cells with normal, impaired and no CAT tailing. ****P < 0.0001; ***P <0.001; ns, not significant. **(D)** Normalized stability measurements for GFP-20 and GFP-40 reporters expressed in *ltn1Δ* cells with variants of *RQC2* chased over 3 hours after introduction of 200 µg/mL cycloheximide.

Figure S3 Folding-induced mechanical forces on the NC regulate CAT tail composition. (A) IBs with model RQC substrates expressed in different genetic backgrounds, including deletion in *VMS1*. The numbers 1 and 2 above *vms1Δ* denote two independent clones that were used in the experiments. **(B)** Cartoon of maltose binding protein (MBP) based RQC substrates with linker of varying lengths (0, 20, 40, 50 and 70 amino acids) between MBP and stalling sequence, analyzed by immunoblotting. **(C)** Stacked bar graph showing the sum of linker and estimated maximum CAT tail length for each RQC substrate in the *ltn1Δ* background. **(D)** Relative aggregation and maximum CAT tail length for each RQC substrate, normalized to the

bioRxiv preprint doi: https://doi.org/10.1101/2024.08.02.606406; this version posted October 14, 2024. The copyright holder for this preprint (which was not certified by peer review) is the author/funder, who has granted bioRxiv a license to display the preprint in perpetuity. It is made available under aCC-BY 4.0 International license.

MBP-6 substrate, in the *ltn1Δ* background. **(E)** Cells expressing GFP-20, 1K-sGFP-40 and 1K-sGFP-20 were grown in media supplemented with DMSO or 1 mM AZC for 3 hours and analyzed by immunoblotting.

Figure S4 Extrinsic mechanical forces on the NC determine CAT tail sequence
(A) Cartoon of ER-directed RQC substrate spGFP-40 alongside the cytosolic variant, with the IBs. **(B)** IBs with model cytosolic and ER directed RQC substrates expressed in *ltn1Δ vms1Δ* background with WT and mutant *RQC2*. **(C)** Schematics showing the RQC role of *CDC48* in different backgrounds. *Top*, in WT cells Cdc48p binds and extracts the ubiquitylated NC from the 60S for proteasomal degradation. *Bottom*, the Cdc48p activity is reduced in the *cdc48-3* background thereby decreasing the pulling force exerted on the NC. **(D)** GFP-20 and GFP-40 reporters expressed in cells with WT and mutant *CDC48*, analyzed by immunoblotting along with the no-CAT tailing (*rqc2Δ*) and no ubiquitylation (*cdc48-3 ltn1Δ*) controls. Long and short CAT tails are marked by hash (#) and asterisk (✻) respectively. Arrowhead (➤) indicates readthrough product formed after bypassing the polyarginine arrest sequence. **(E)** GFP-40 reporter expressed in *ump1Δ* cells, compared with WT, non-ubiquitylated (*ltn1Δ*), and non-CAT-tailed (*rqc2Δ*) controls

bioRxiv preprint doi: https://doi.org/10.1101/2024.08.02.606406; this version posted October 14, 2024. The copyright holder for this preprint (which was not certified by peer review) is the author/funder, who has granted bioRxiv a license to display the preprint in perpetuity. It is made available under aCC-BY 4.0 International license.

Figure S7 CAT tails regulate NC retention and release (A) *Left,* **Fluorescent polysome profiling of** WT cells after 8 hours of cycloheximide treatment, harboring no plasmid, showed a background fluorescence peak at 40S position; Fluorescent polysome profiling of *ltn1Δ* (*center*) and *ltn1Δ rqc2*-D98A (*right*) cells expressing GFP-40-RRR-stop plasmid as a control. **(B)** Fluorescent polysome profiling of GFP-40/*ltn1Δ* **(***left***)** and GFP-40/*ltn1Δrqc2*-D98A (*right*) in a 'high salt' buffer containing 500 mM KCl. Dotted lines in the traces indicate that profiles were collected under high salt conditions. **(C)** GFP-S4(K) and **(D)** GFP-S4(D) reporters expressed in *ltn1Δ* cells with WT *RQC2*, *rqc2*-D9A and *rqc2*-D98A, were analyzed by fluorescent polysome profiling. **(E)** Schematic representation of models for amino acid specificity in CAT tailing. *Top*, *'Energy Barrier'* model. In the absence of a pulling force on the NC, the P-site tRNA has higher degrees of freedom, allowing it to incorporate both Ala and Thr into the CAT tail. This increased entropy of the P-site tRNA helps overcome the higher activation energy barrier for Thr incorporation. When a pulling force is applied to the NC, it restricts the P-site tRNA's mobility and lowers its entropy. Under these conditions, Ala is selectively incorporated due to its lower activation energy barrier for peptide bond formation, allowing it to more easily accommodate the restricted state of the peptidyl transferase center. *Bottom,* '*tRNA recruitment* model'. Pulling forces regulate the recruitment of charged tRNAs at the A-site via allosteric interactions with Rqc2p. The NFACT-N/R and NFACT-C domains stabilize the anticodon arm of P-site tRNA, whereas the NFACT-C domain and 60S facing side of CCM loop stabilize the anticodon arm of A-site tRNAs. The NFACT-N domain specifically recruits Ala-tRNA^{AGC} and Thr-tRNA^{AGT} and delivers them to the 60S A-site. In the absence of a pulling force, recruitment of both Ala- and Thr-tRNA is favored. The presence of pulling force reduces the affinity of NFACT-N domain for Thr-tRNA, thereby favoring the recruitment of Ala-tRNA.

bioRxiv preprint doi: https://doi.org/10.1101/2024.08.02.606406; this version posted October 14, 2024. The copyright holder for this preprint (which was not certified by peer review) is the author/funder, who has granted bioRxiv a license to display the preprint in perpetuity. It is made available under aCC-BY 4.0 International license.

Fig. S1: CAT tails have diverse composition, degradation, and aggregation propensities

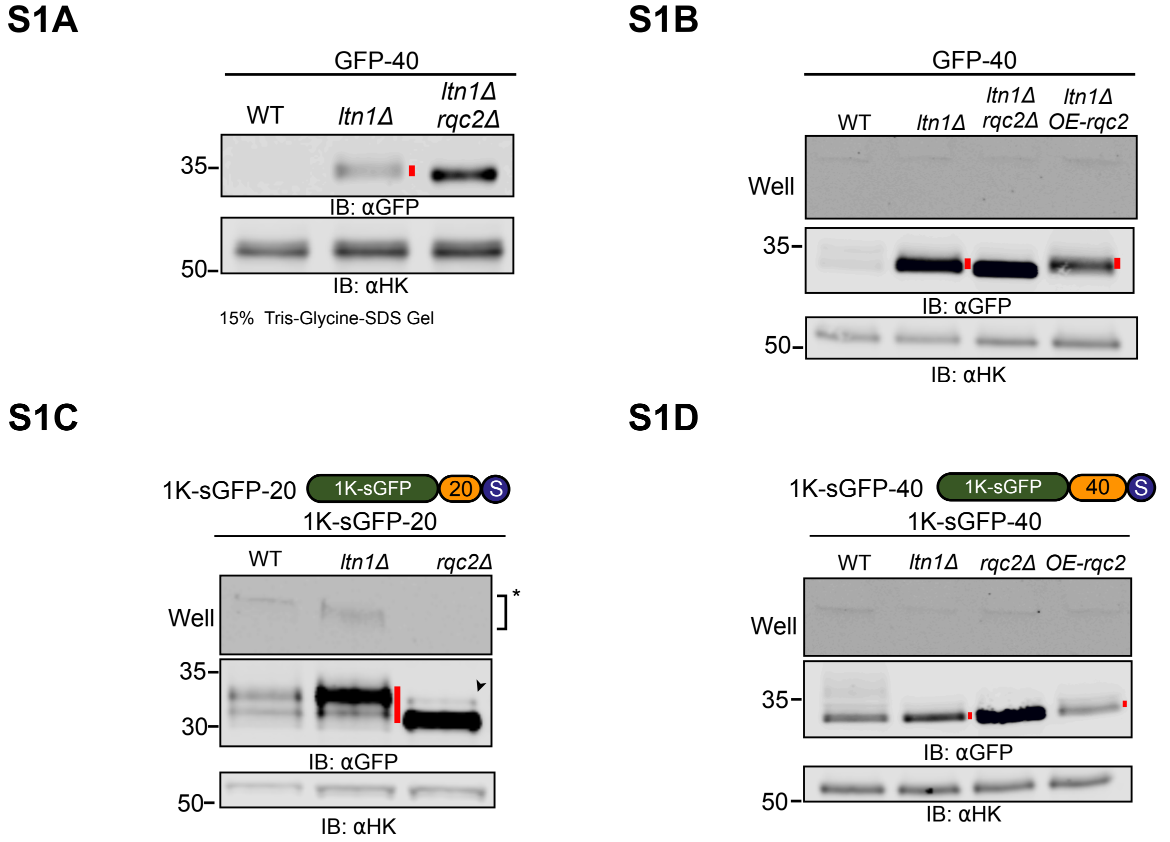

bioRxiv preprint doi: https://doi.org/10.1101/2024.08.02.606406; this version posted October 14, 2024. The copyright holder for this preprint (which was not certified by peer review) is the author/funder, who has granted bioRxiv a license to display the preprint in perpetuity. It is made available under aCC-BY 4.0 International license.

Fig. S2: CAT tail sequence determines degradation and aggregation

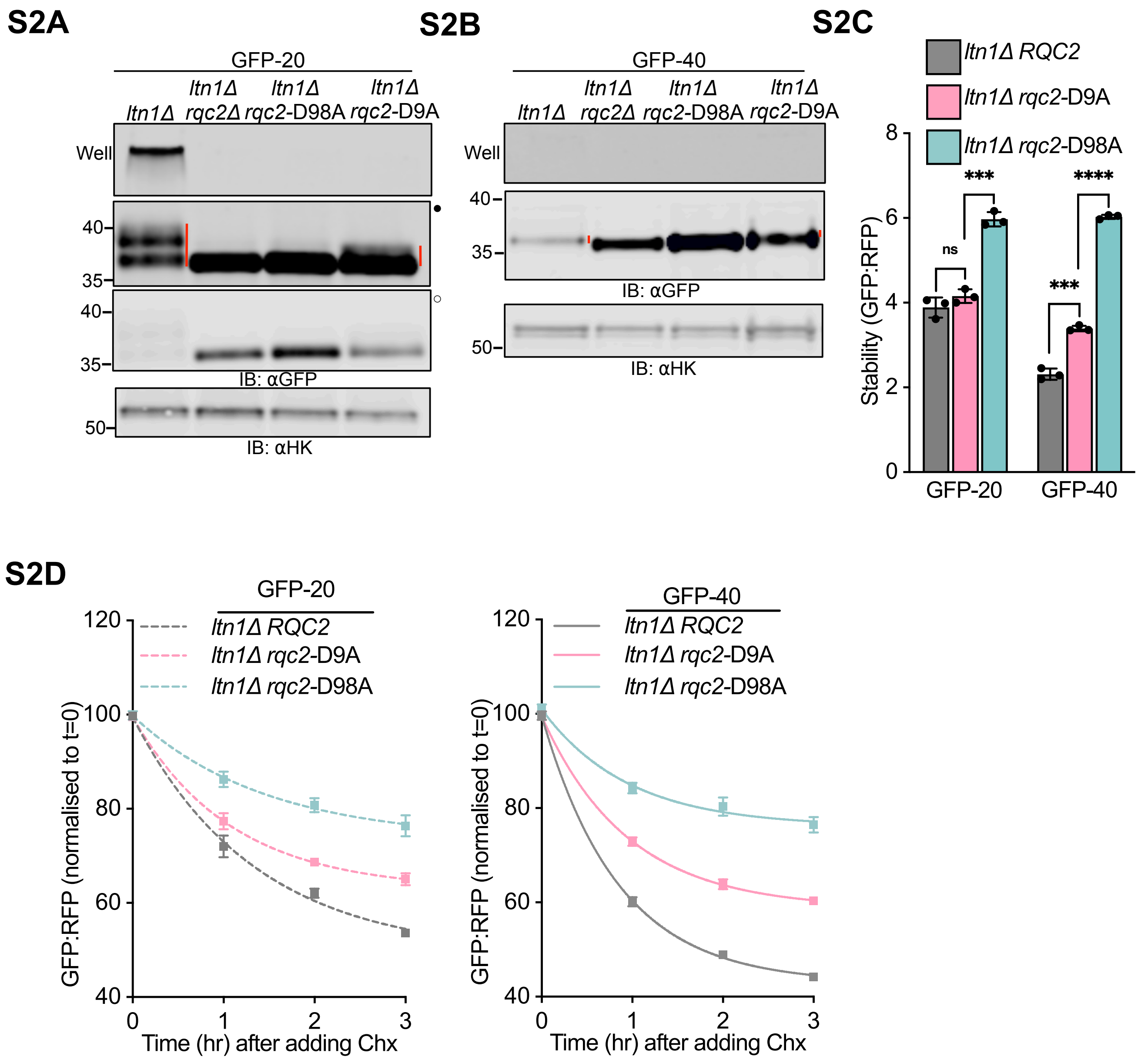

bioRxiv preprint doi: https://doi.org/10.1101/2024.08.02.606406; this version posted October 14, 2024. The copyright holder for this preprint (which was not certified by peer review) is the author/funder, who has granted bioRxiv a license to display the preprint in perpetuity. It is made available under aCC-BY 4.0 International license.

Fig. S3: Folding-induced mechanical forces on the NC determine CAT tail composition

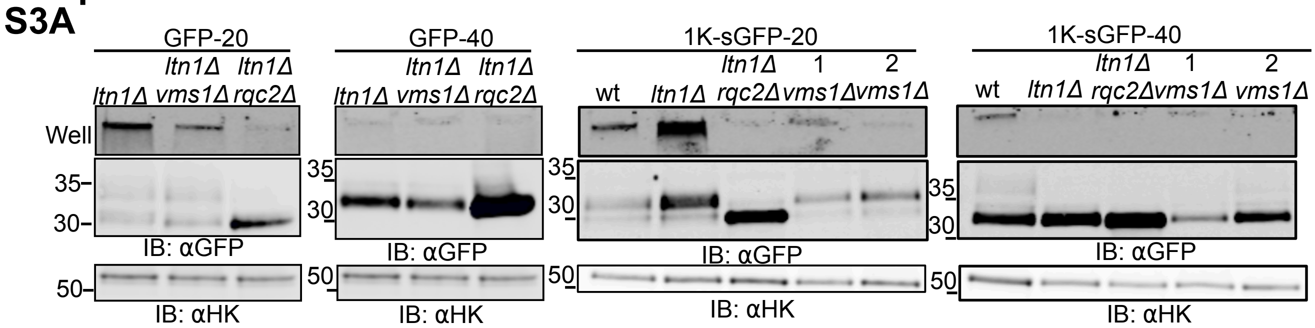

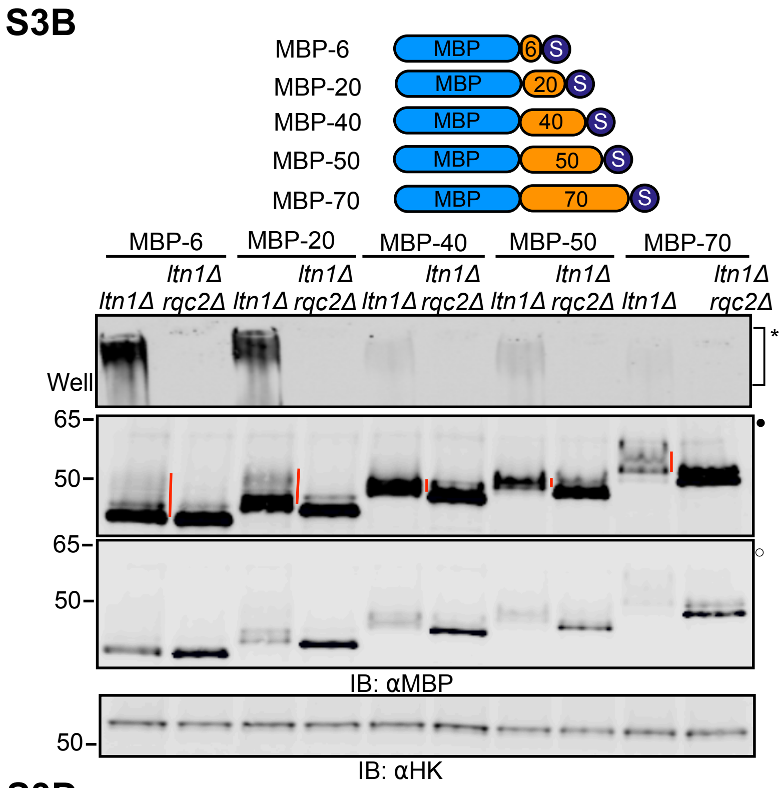

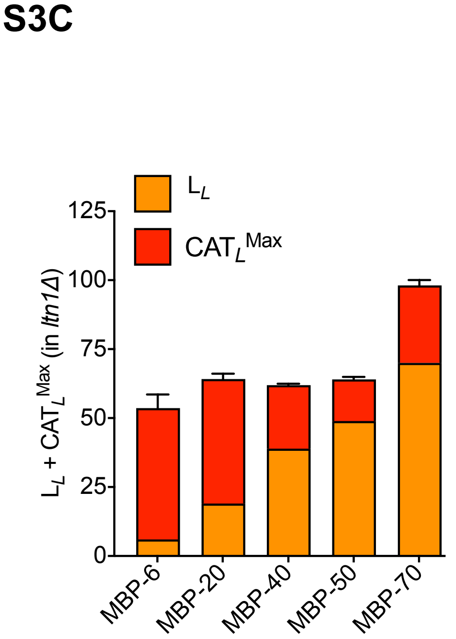

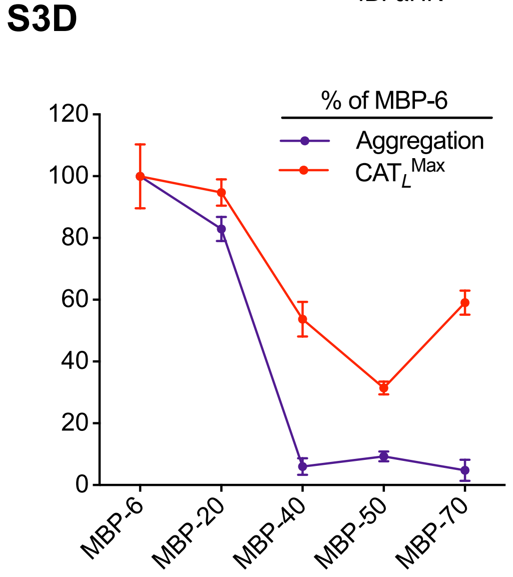

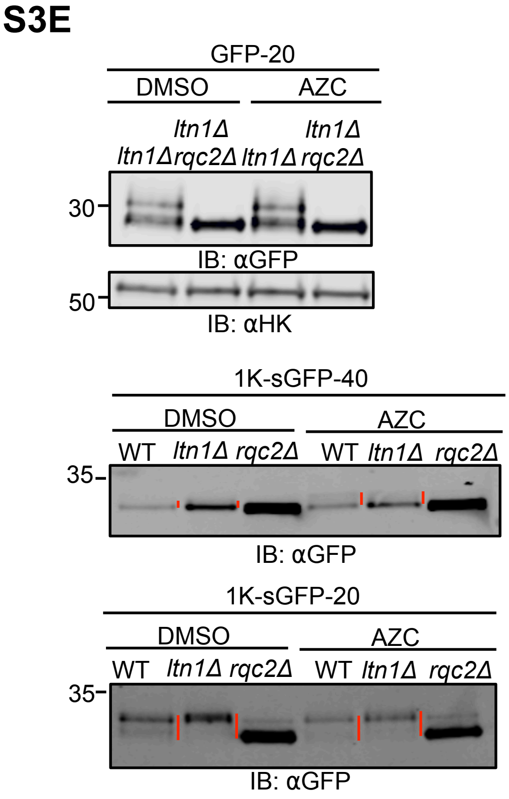

bioRxiv preprint doi: https://doi.org/10.1101/2024.08.02.606406; this version posted October 14, 2024. The copyright holder for this preprint (which was not certified by peer review) is the author/funder, who has granted bioRxiv a license to display the preprint in perpetuity. It is made available under aCC-BY 4.0 International license.

Fig. S4: Extrinsic mechanical forces on the NC determine CAT tail sequence

S4A

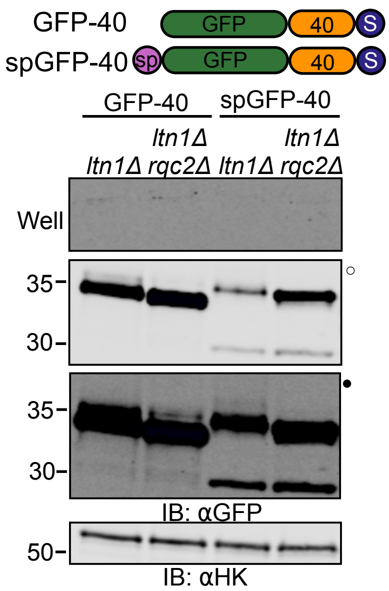

S4B

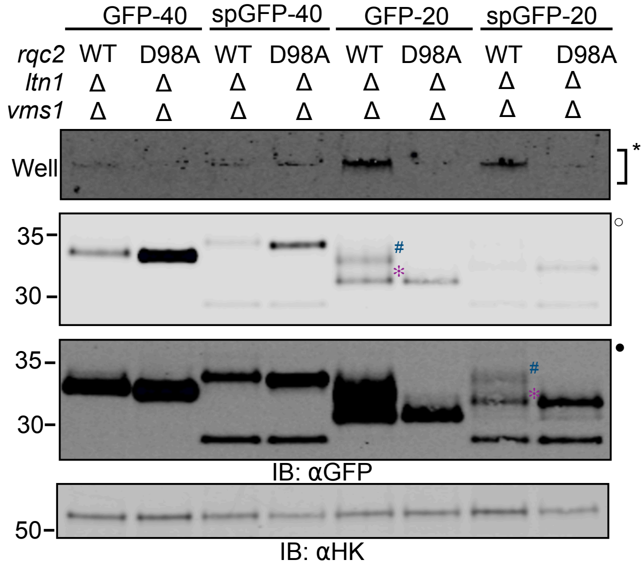

S4C

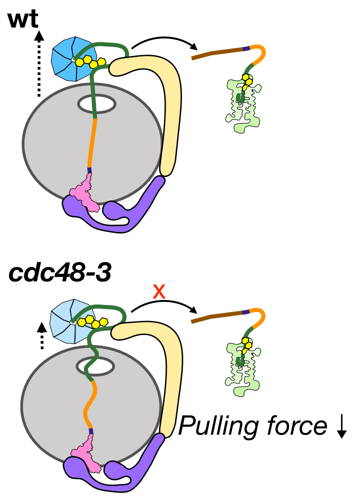

S4D

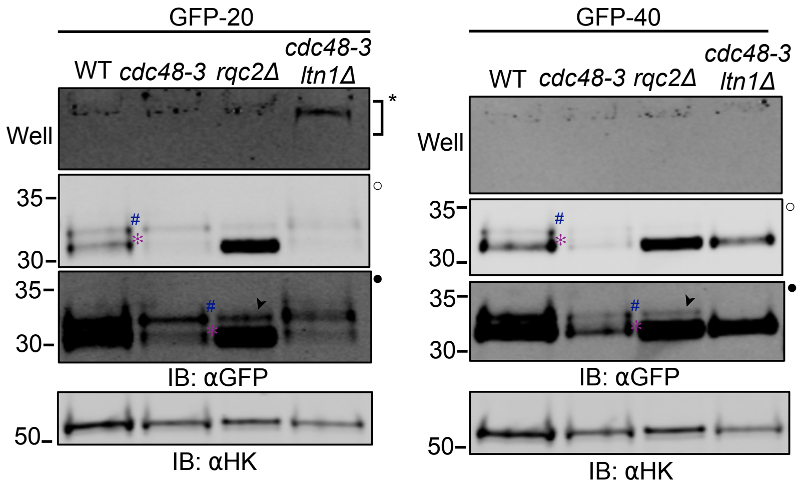

S4E

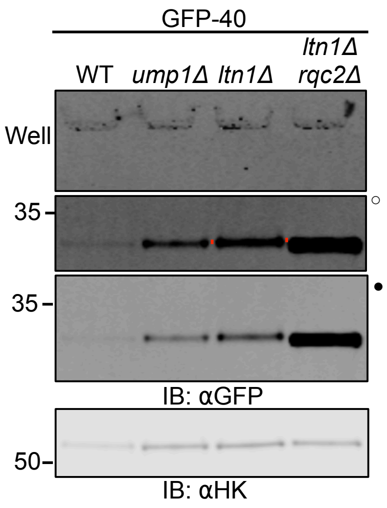

bioRxiv preprint doi: https://doi.org/10.1101/2024.08.02.606406; this version posted October 14, 2024. The copyright holder for this preprint (which was not certified by peer review) is the author/funder, who has granted bioRxiv a license to display the preprint in perpetuity. It is made available under aCC-BY 4.0 International license.

Fig. S7: CAT tails regulate NC retention and release

S7A

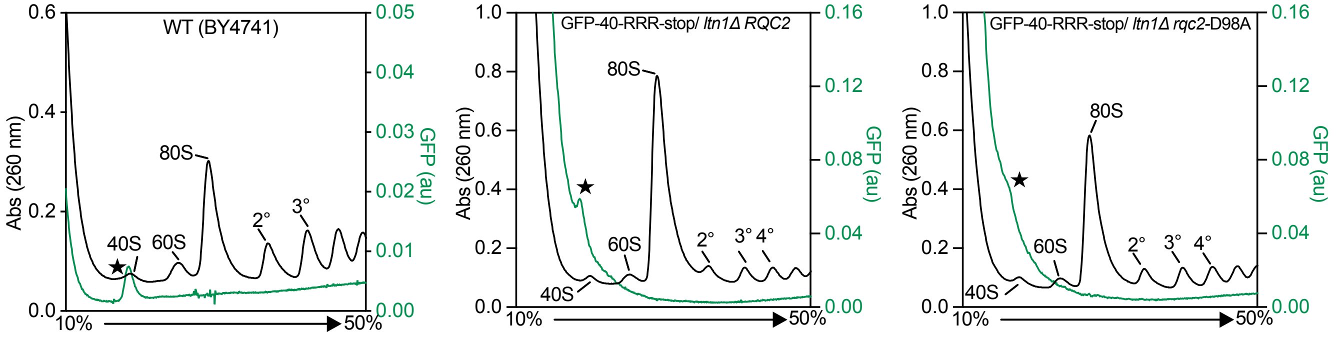

S7B

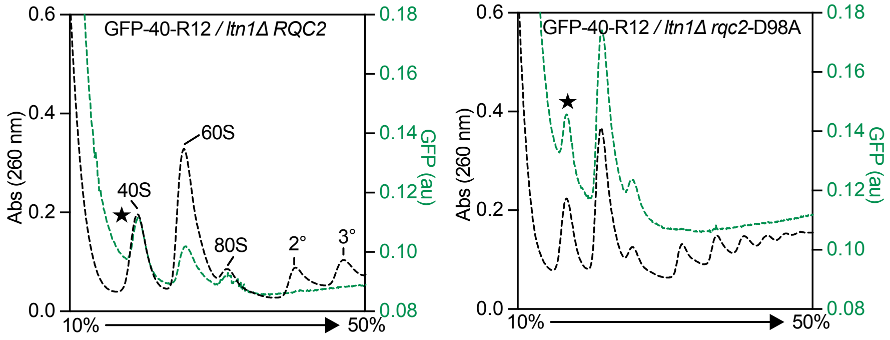

S7C

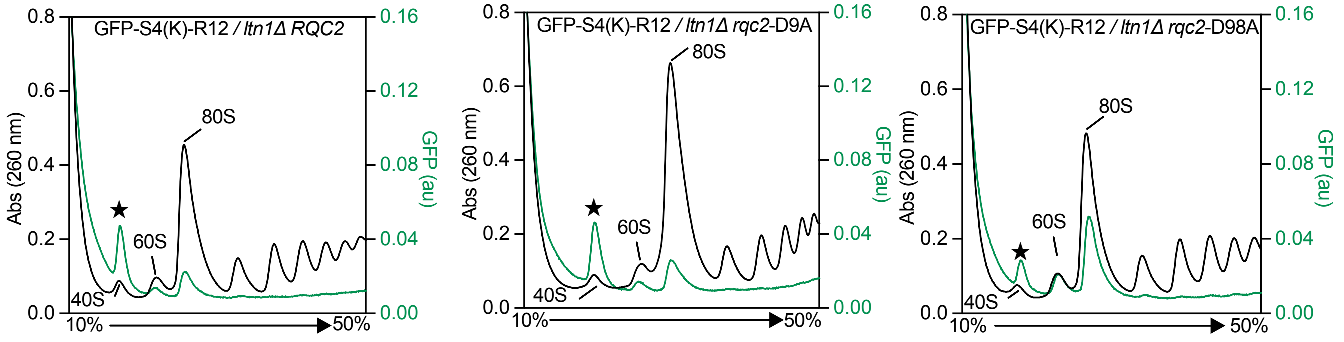

S7D

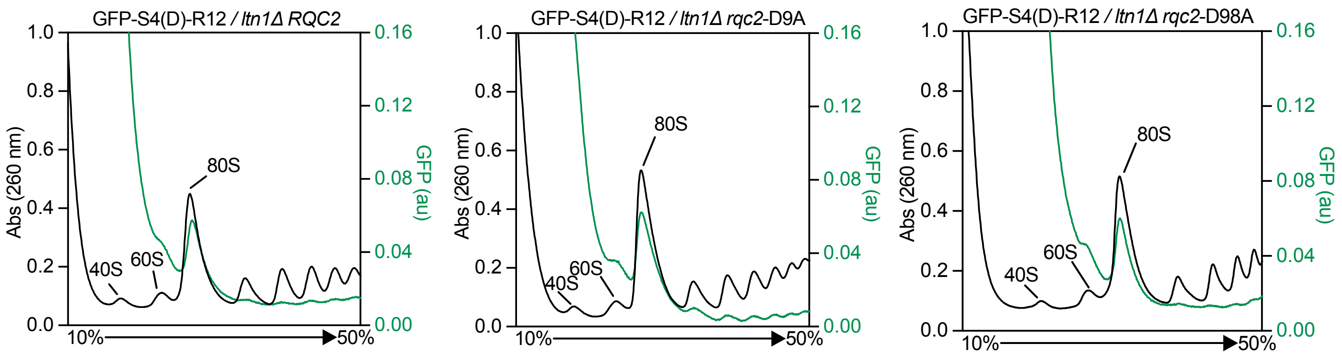

bioRxiv preprint doi: https://doi.org/10.1101/2024.08.02.606406; this version posted October 14, 2024. The copyright holder for this preprint (which was not certified by peer review) is the author/funder, who has granted bioRxiv a license to display the preprint in perpetuity. It is made available under aCC-BY 4.0 International license.

Fig. S7: CAT tails regulate NC retention and release

S7E

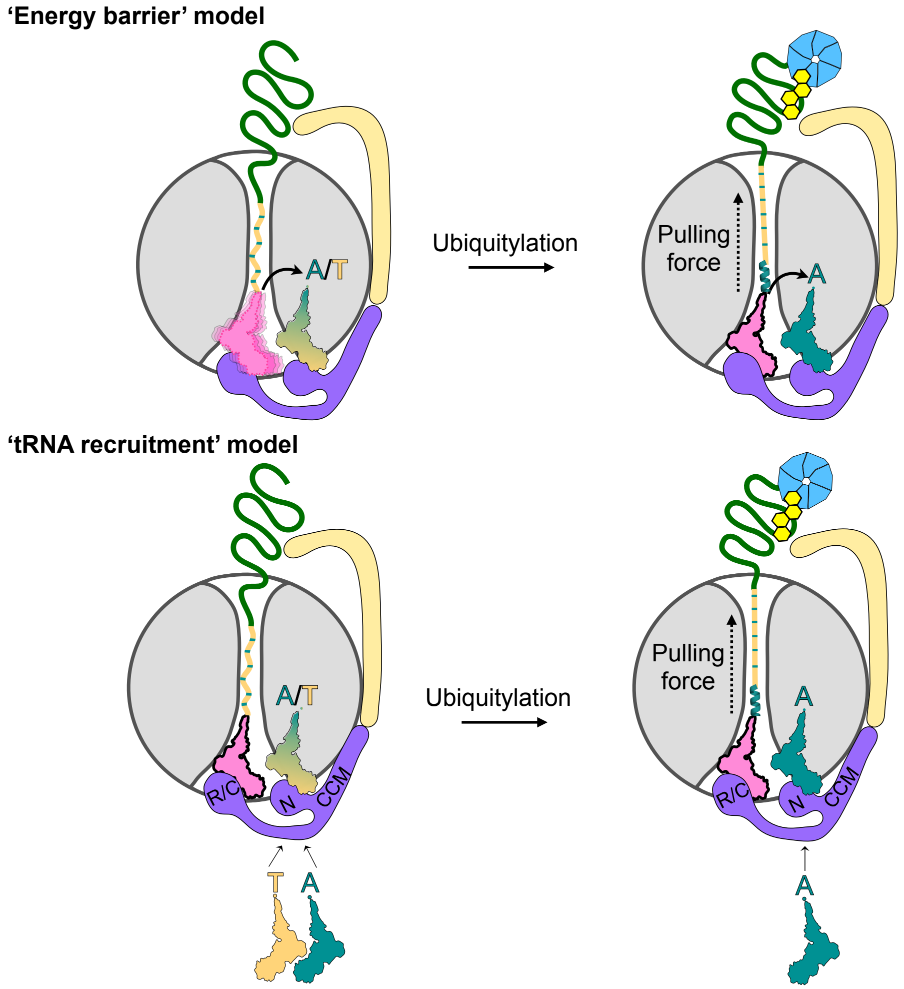

Supplement: Supplement 2 [file NIHPP2024.08.02.606406v2-supplement-2.pdf]
