# Supplementary material for: Mechanochemical forces regulate the composition and function of CAT tails
Source: bioRxiv. 2024 Aug 4:2024.08.02.606406. Preprint. [Version 1] doi: 10.1101/2024.08.02.606406 (PMC11312545; doi:10.1101/2024.08.02.606406)

bioRxiv preprint doi: https://doi.org/10.1101/2024.08.02.606406; this version posted August 4, 2024. The copyright holder for this preprint (which was not certified by peer review) is the author/funder, who has granted bioRxiv a license to display the preprint in perpetuity. It is made available under aCC-BY 4.0 International license.

Figure S1 CAT tails have diverse composition, degradation, and aggregation propensities. (A-D) Whole cell IBs of lysates containing model RQC substrates expressed in different strains and gel densities. Cartoon of model RQC substrates with single lysine superfolder GFP (1K-sGFP) indicated where applicable. CAT tails are marked by a red line. Arrowhead (➤) indicates readthrough product formed after bypassing the polyarginine arrest sequence.

bioRxiv preprint doi: https://doi.org/10.1101/2024.08.02.606406; this version posted August 4, 2024. The copyright holder for this preprint (which was not certified by peer review) is the author/funder, who has granted bioRxiv a license to display the preprint in perpetuity. It is made available under aCC-BY 4.0 International license.

Figure S2 CAT tail sequence determines degradation and aggregation. (A-B) Whole cell immunoblots of GFP-20 and GFP-40 expressed in *ltn1Δ* cells with varying CAT tailing capabilities: normal CAT tailing (WT, lane 1), no CAT tailing (*rqc2Δ,* lane 2; *rqc2*-D98A, lane 3), and impaired CAT tailing (*rqc2*-D9A, lane 4). **(C)** Mean stability measurements for GFP-20 and GFP-40 substrates in cells with normal, impaired and no CAT tailing. ****P < 0.0001; ***P <0.001; ns, not significant. **(D)** Normalized stability measurements for GFP-20 and GFP-40 reporters expressed in *ltn1Δ* cells with variants of *RQC2* chased over 3 hours after introduction of 200 μg/mL cycloheximide.

Figure S3 Folding-induced mechanical forces on the NC regulate CAT tail composition. (A) IBs with model RQC substrates expressed in different genetic backgrounds, including deletion in *VMS1*. The numbers 1 and 2 above *vms1Δ* denote two independent clones that were used in the experiments. **(B)** Cartoon of maltose binding protein (MBP) based RQC substrates with linker of varying lengths (0, 20, 40, 50 and 70 amino acids) between MBP and stalling sequence, analyzed by immunoblotting. **(C)** Stacked bar graph showing the sum of linker and estimated maximum CAT tail length for each RQC substrate in the *ltn1Δ* background. **(D)** Relative aggregation and maximum CAT tail length for each RQC substrate, normalized to the

bioRxiv preprint doi: https://doi.org/10.1101/2024.08.02.606406; this version posted August 4, 2024. The copyright holder for this preprint (which was not certified by peer review) is the author/funder, who has granted bioRxiv a license to display the preprint in perpetuity. It is made available under aCC-BY 4.0 International license.

MBP-6 substrate, in the *ltn1Δ* background. **(E)** Cells expressing GFP-20, 1K-sGFP-40 and 1K-sGFP-20 were grown in media supplemented with DMSO or 1 mM AZC for 3 hours and analyzed by immunoblotting.

Figure S4 Extrinsic mechanical forces on the NC determine CAT tail sequence
(A) Cartoon of ER-directed RQC substrate spGFP-40 alongside the cytosolic variant, with the IBs. **(B)** IBs with model cytosolic and ER directed RQC substrates expressed in *ltn1Δ vms1Δ* background with WT and mutant *RQC2*. **(C)** Schematics showing the RQC role of *CDC48* in different backgrounds. *Top*, in WT cells Cdc48p binds and extracts the ubiquitylated NC from the 60S for proteasomal degradation. *Bottom*, the Cdc48p activity is reduced in the *cdc48-3* background thereby decreasing the pulling force exerted on the NC. **(D)** GFP-20 and GFP-40 reporters expressed in cells with WT and mutant *CDC48*, analyzed by immunoblotting along with the no-CAT tailing (*rqc2Δ*) and no ubiquitylation (*cdc48-3 ltn1Δ*) controls. Long and short CAT tails are marked by hash (#) and asterisk (✻) respectively. Arrowhead (➤) indicates readthrough product formed after bypassing the polyarginine arrest sequence. **(E)** GFP-40 reporter expressed in *ump1Δ* cells, compared with WT, non-ubiquitylated (*ltn1Δ*), and non-CAT-tailed (*rqc2Δ*) controls

bioRxiv preprint doi: https://doi.org/10.1101/2024.08.02.606406; this version posted August 4, 2024. The copyright holder for this preprint (which was not certified by peer review) is the author/funder, who has granted bioRxiv a license to display the preprint in perpetuity. It is made available under aCC-BY 4.0 International license.

Figure S7 CAT tails regulate NC retention and release (A) WT cells, harboring no plasmid, showed a background fluorescence peak at 40S position. **(B-C)** Fluorescent polysome profiling of *ltn1Δ* **(B)** and *ltn1Δ rqc2*-D98A **(C)** cells expressing GFP-RRR-stop as a control. **(D-E)** Fluorescent polysome profiling of GFP-40/*ltn1Δ* **(D)** and GFP-40/*ltn1Δrqc2*-D98A **(E)** in a ‘high

bioRxiv preprint doi: https://doi.org/10.1101/2024.08.02.606406; this version posted August 4, 2024. The copyright holder for this preprint (which was not certified by peer review) is the author/funder, who has granted bioRxiv a license to display the preprint in perpetuity. It is made available under aCC-BY 4.0 International license.

Fig. S1: CAT tails have diverse composition, degradation, and aggregation propensities

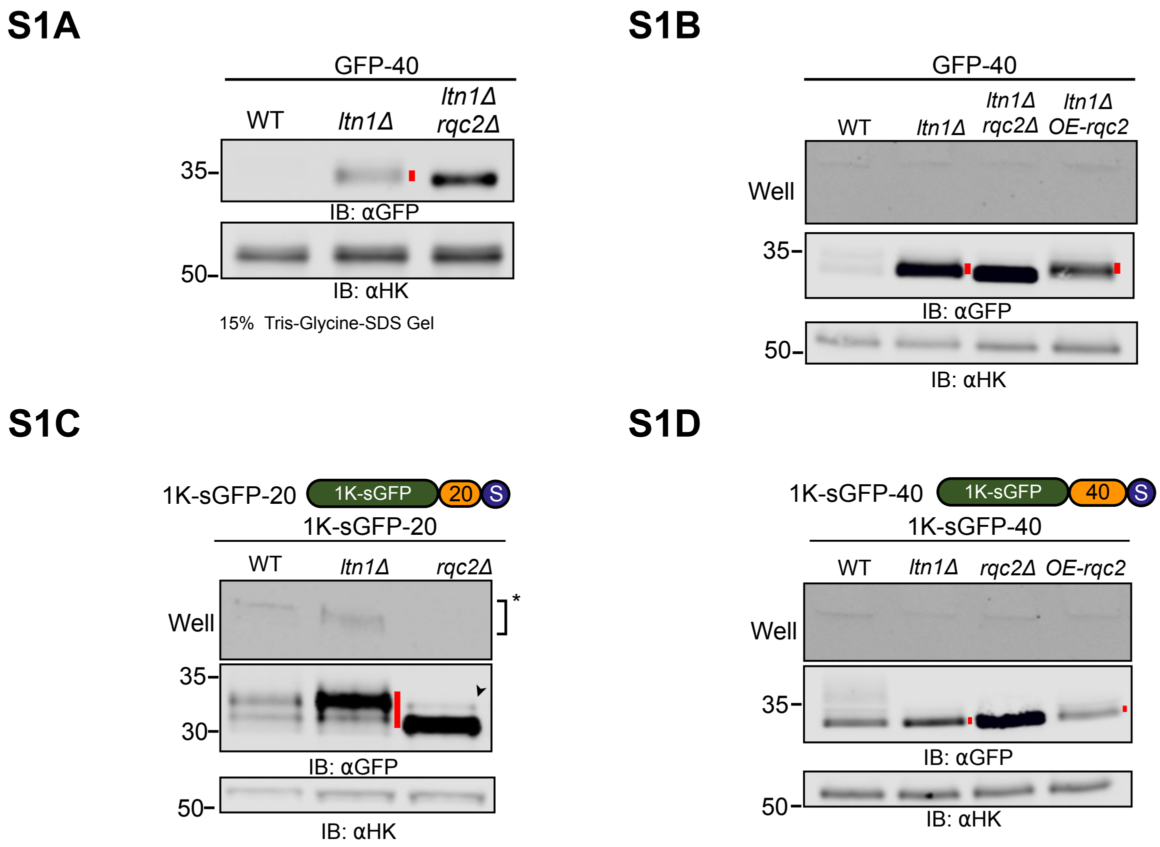

bioRxiv preprint doi: https://doi.org/10.1101/2024.08.02.606406; this version posted August 4, 2024. The copyright holder for this preprint (which was not certified by peer review) is the author/funder, who has granted bioRxiv a license to display the preprint in perpetuity. It is made available under aCC-BY 4.0 International license.

Fig. S2: CAT tail sequence determines degradation and aggregation

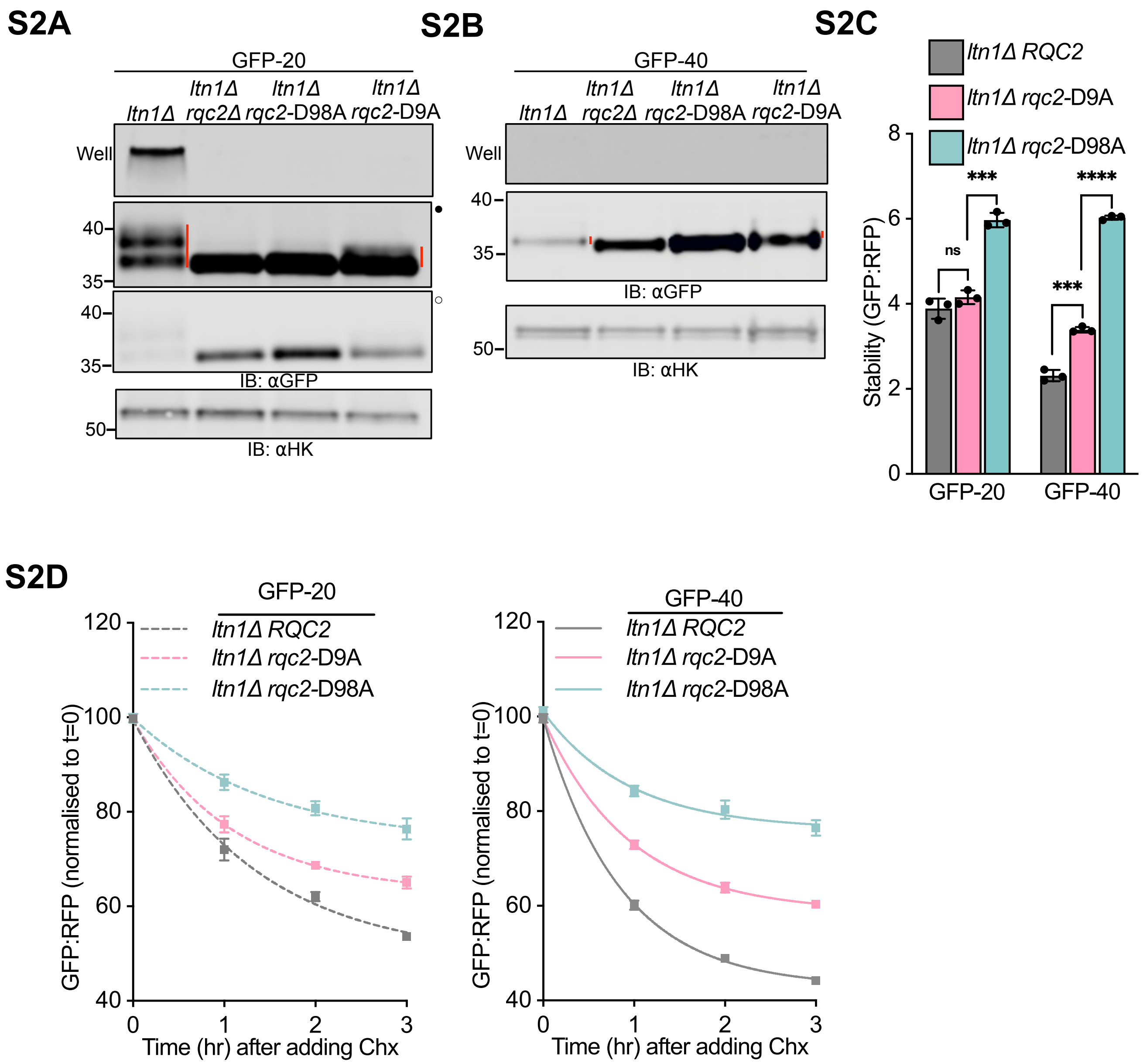

bioRxiv preprint doi: https://doi.org/10.1101/2024.08.02.606406; this version posted August 4, 2024. The copyright holder for this preprint (which was not certified by peer review) is the author/funder, who has granted bioRxiv a license to display the preprint in perpetuity. It is made available under aCC-BY 4.0 International license.

Fig. S3: Folding-induced mechanical forces on the NC determine CAT tail composition

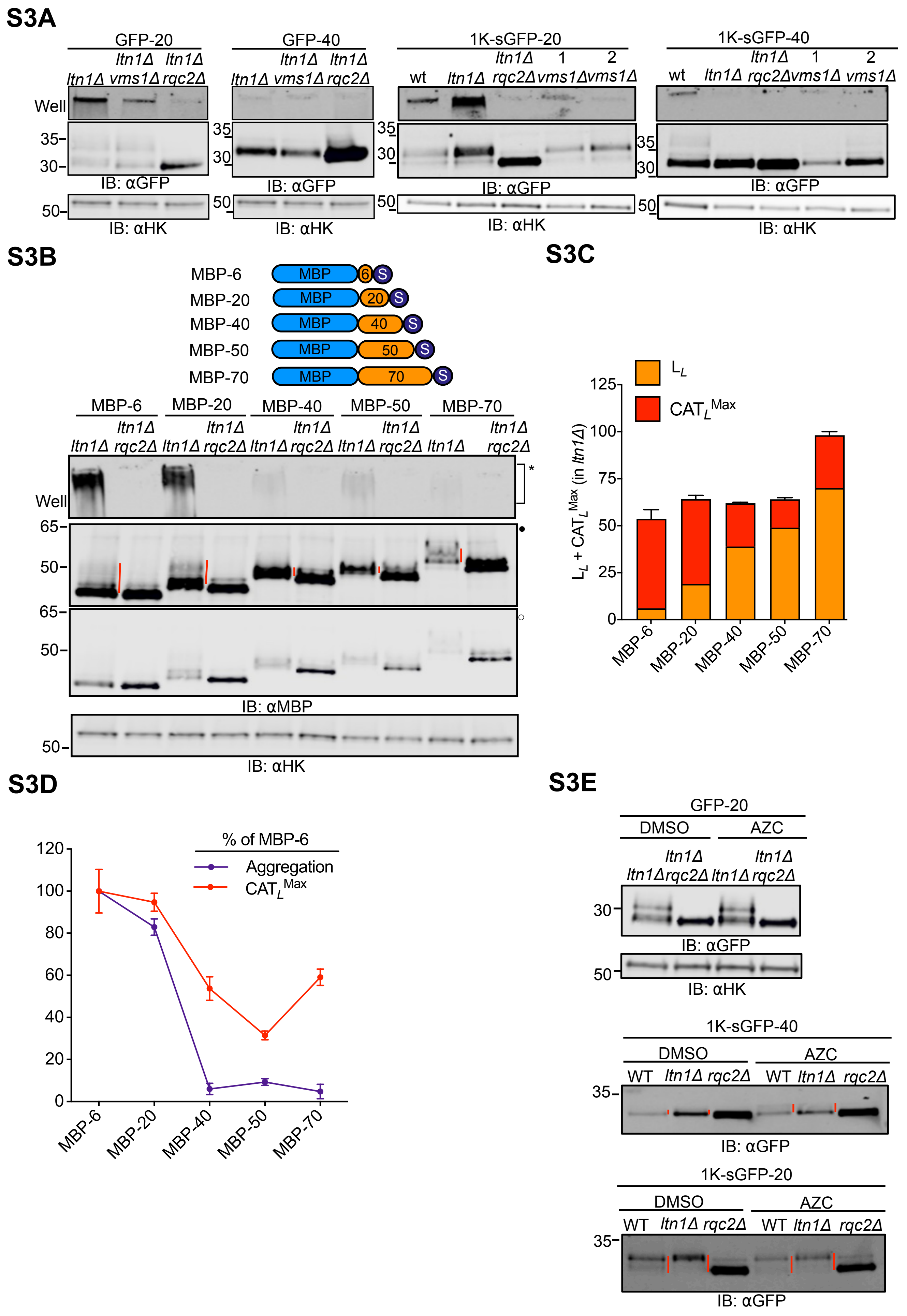

bioRxiv preprint doi: https://doi.org/10.1101/2024.08.02.606406; this version posted August 4, 2024. The copyright holder for this preprint (which was not certified by peer review) is the author/funder, who has granted bioRxiv a license to display the preprint in perpetuity. It is made available under aCC-BY 4.0 International license.

Fig. S4: Extrinsic mechanical forces on the NC determine CAT tail sequence

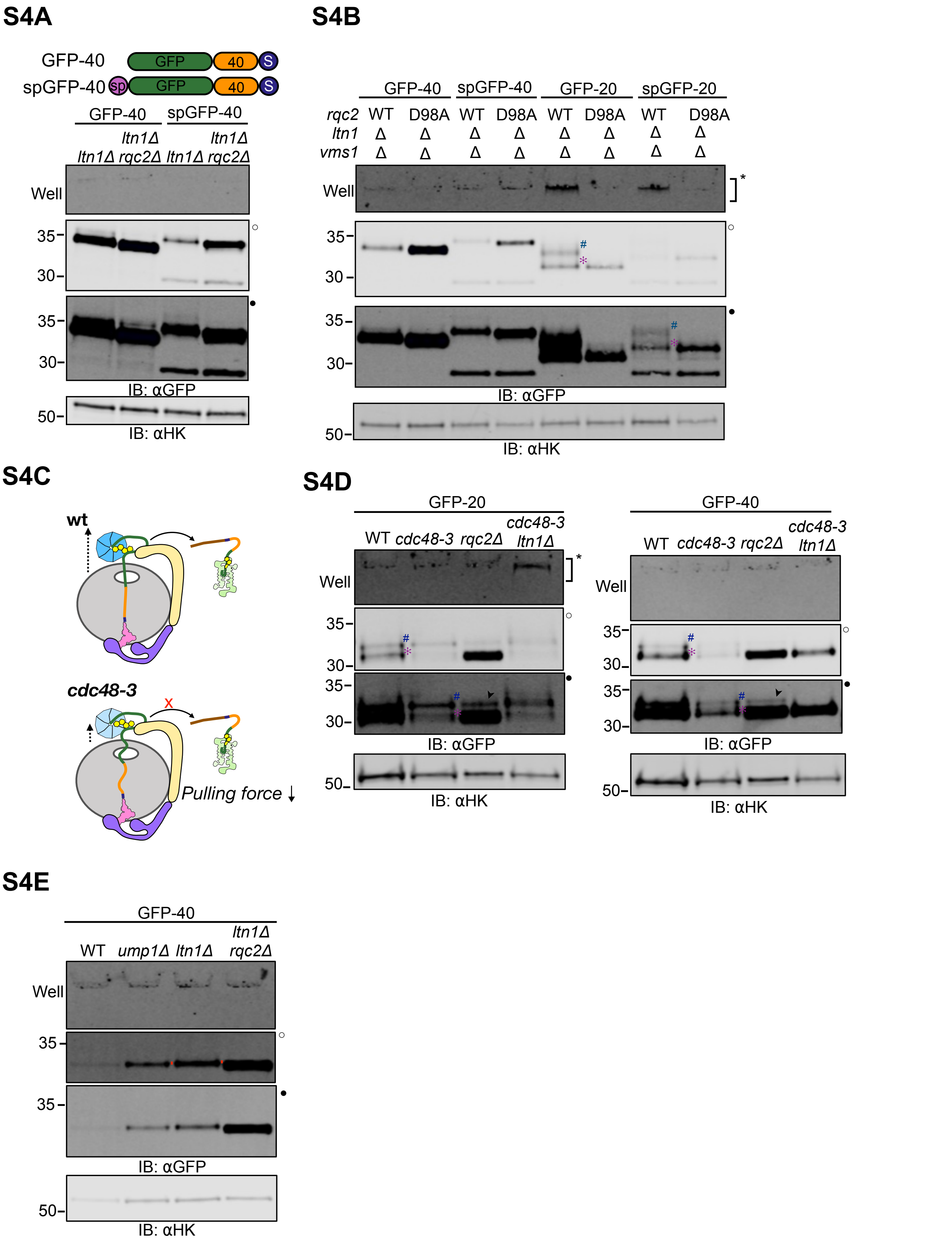

bioRxiv preprint doi: https://doi.org/10.1101/2024.08.02.606406; this version posted August 4, 2024. The copyright holder for this preprint (which was not certified by peer review) is the author/funder, who has granted bioRxiv a license to display the preprint in perpetuity. It is made available under aCC-BY 4.0 International license.

Fig. S7: CAT tails regulate NC retention and release

S7A

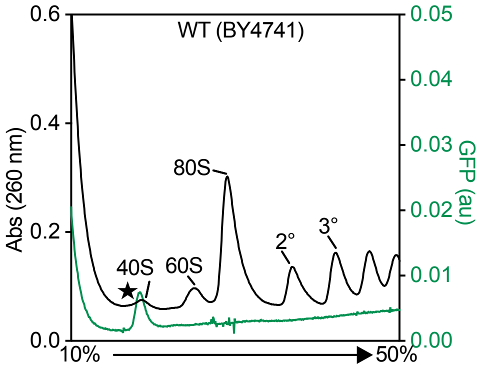

S7B

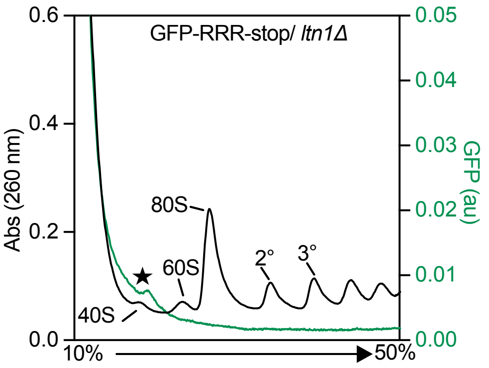

S7C

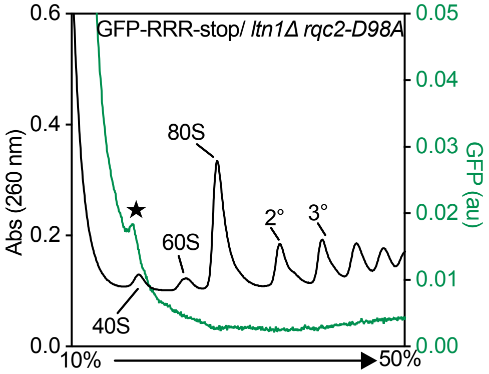

S7D

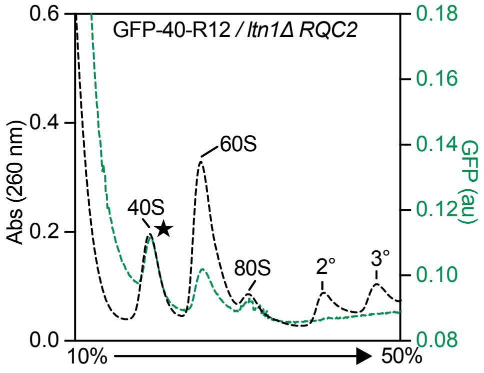

S7E

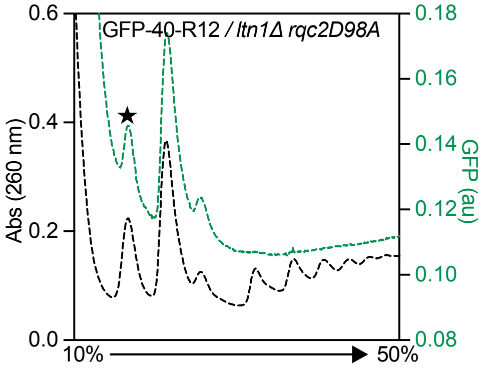

bioRxiv preprint doi: https://doi.org/10.1101/2024.08.02.606406; this version posted August 4, 2024. The copyright holder for this preprint (which was not certified by peer review) is the author/funder, who has granted bioRxiv a license to display the preprint in perpetuity. It is made available under aCC-BY 4.0 International license.

Fig. S7: CAT tails regulate NC retention and release

S7F

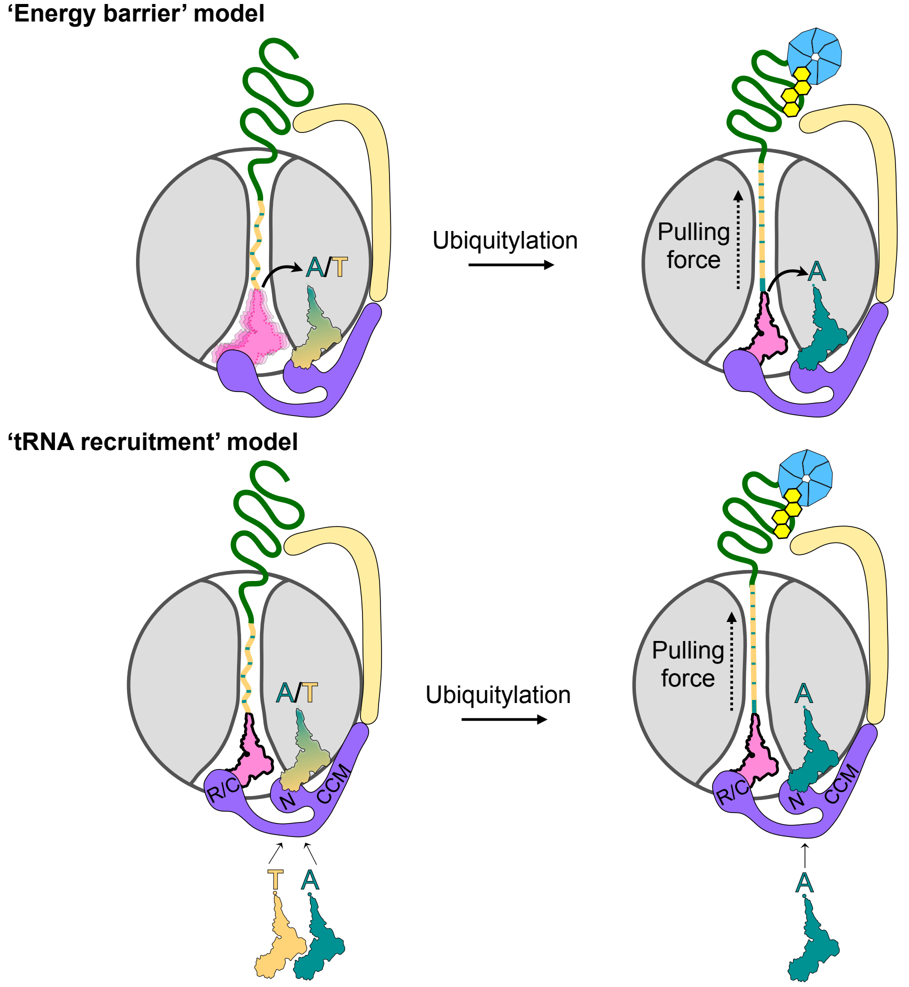

Supplement: Supplementary file 1 [file NIHPP2024.08.02.606406v1-supplement-1.pdf]
